# Role of TNF-α genetic variants in coinfection with *Helicobacter pylori* and *the Entamoeba* complex*:* a cross-sectional study

**DOI:** 10.1186/s12866-026-04964-2

**Published:** 2026-04-16

**Authors:** Asmaa Ibrahim, Moamen A. I. Mazen, Doaa Eltaweel, Soha Mahmoud Abd El Salam, Abeer Abdelkader, Rasha Elgamal, Maysa I. Farghly, Mohamed W. Saleh, Tarek Mostafa Ibrahim Mohamed AbdElRahim, Hany Gamal Abozaid Metwaly, Eman Hany Elsebaie, Mohamed Medhat, Manar Ezzelarab Ramadan

**Affiliations:** 1 Department of Molecular Biology, Faculty of Biotechnology, University of Sadat City, El Sadat City, Egypt; 2 Department of Medical Parasitology, Faculty of Medicine, Cairo University (Laboratory of Molecular Medical Parasitology, LMMP), Cairo, Egypt; 3 Department of Medical Parasitology, Faculty of Medicine, Al-Azhar University, Assiut, Egypt; 4 Lecture of Medical Microbiology and Immunology Department, Faculty of Medicine, Cairo University, Cairo, Egypt; 5 Department of Microbiology and Immunology, Faculty of Medicine, Suez University, Suez, Egypt; 6 Lecturer of Medical Biochemistry&Molecular Biology Department, Faculty of Medicine, Cairo University, Cairo, Egypt; 7 Clinical Pathology Department, Faculty of Medicine, Suez University, Suez, Egypt; 8 Lecturer of Internal Medicine, Faculty of Medicine, Suez University, Suez, Egypt; 9 Lecturer of Gastroenterology, Hepatology, and Infectious Diseases, Faculty of Medicine, Suez University, Suez, Egypt; 10 Lecturer of Internal Medicine, Faculty of Medicine, Al-Azhar University, Assiut, Egypt; 11 Public Health and Community Medicine Department, Faculty of Medicine, Cairo University, Cairo, Egypt; 12 Lecturer of Infectious Diseases, Gastroenterology and Hepatology, Suez University, Suez, Egypt; 13 Department of Medical Parasitology, Faculty of Medicine, Suez University, P.O. Box:43221, Suez, Egypt

**Keywords:** *H. pylori*, *E. complex*, Molecular identification, TNF-α gene polymorphism, Co-infection

## Abstract

**Background:**

*Helicobacter pylori* and *Entamoeba histolytica* are among the most common gastrointestinal pathogens worldwide, particularly in developing countries where sanitation conditions favor transmission. Co-infection by these organisms may influence disease manifestation and host immune response. This study aimed to determine the prevalence and molecular characterization of *Entamoeba* species and *Helicobacter pylori* among patients with gastrointestinal symptoms attending Kasr El-Aini hospital. It also investigated the association of tumor necrosis factor-alpha (TNF-α) gene polymorphisms at positions − 1031 T/C and − 308G/A with susceptibility or protection against these infections.

**Results:**

A total of 372 stool samples were collected and classified into four groups: *Helicobacter pylori*-only, *Entamoeba*-only, co-infected, and control. Controls were recruited from the same clinical setting and confirmed negative for both *Helicobacter pylori* and *Entamoeba histolytica* by molecular assays. Microscopic examination was first performed for parasitic detection, followed by DNA extraction and polymerase chain reaction (PCR) for species identification of *Entamoeba dispar*, *Entamoeba moshkovskii*, *Entamoeba histolytica*, and *Helicobacter pylori*. Among 93 samples positive for the *Entamoeba* complex, *Entamoeba moshkovskii* was the predominant species (53.8%), followed by *Entamoeba. dispar* (29.0%) and *Entamoeba histolytica* (17.2%). Mixed infections were observed in 19 cases. Sociodemographic factors, including age, sex, residence, water source, and animal contact, were significantly associated with the presence of *Entamoeba* species. Among 93 *Helicobacter pylori*-positive participants, 44.1% carried the cytotoxin-associated gene A (CagA) virulence factor. Sex and tap water consumption showed significant associations with *Helicobacter pylori* infection and co-infection. Genotyping analysis revealed that the TNF-α − 1031 T/C TT genotype and T allele were more frequent in healthy controls, suggesting a protective role, while no significant difference was observed for the − 308G/A polymorphism.

**Conclusions:**

This study highlights a notable prevalence of *Entamoeba moshkovskii* among the *Entamoeba complex* and a considerable rate of co-infection with *Helicobacter pylori*. Environmental and host factors, particularly water source and sex, play important roles in infection dynamics. The TNF-α − 1031C allele appears to confer protection against gastrointestinal infection. These findings emphasize the need for improved diagnostic screening and public health measures to limit transmission and co-infection in endemic areas.

**Supplementary Information:**

The online version contains supplementary material available at 10.1186/s12866-026-04964-2.

## Introduction

*Entamoeba histolytica* (*E.histolytica*) is the primary causative protozoan of human amebiasis; its clinical manifestations range from asymptomatic colonization to a severe form of intestinal disease and may include life-threatening extraintestinal complications such as hepatic abscesses. Globally, amoebic infection affects nearly 50 million people worldwide, contributing to approximately 2.24 million disability-adjusted life years (DALYs) and more than one hundred thousand fatalities per year [1]. The *Entamoeba* complex, which mostly includes *Entamoeba histolytica*, *Entamoeba dispar*, and *Entamoeba moshkovskii,* consists of morphologically indistinguishable species under light microscopy [2]. *E. dispar* is generally considered non-pathogenic, whereas *E. moshkovskii*, once regarded as non-pathogenic, has increasingly been associated with gastrointestinal symptoms in several studies, suggesting potential pathogenicity under certain conditions [3]. Due to their morphological similarity under light microscopy, these species have historically been misidentified as *E. histolytica*, highlighting the role of molecular diagnostics for accurate differentiation [4]. The development of amoebic infection requires a balance between parasite virulence and host immunity. Key stages in amoebic infection include mucosal barrier disruption, epithelial adhesion, tissue damage, and potential dissemination to extraintestinal sites. Nevertheless, the exact molecular mechanisms underlying tissue invasion by pathogenic amoebae are not yet fully elucidated. Once tissue invasion occurs, the host immune system mounts a response aimed at controlling and eliminating the parasite [5, 6].

*Helicobacter pylori (H.pylori)* is a human-infecting gram-negative bacterium that colonizes the gastric mucosa and is linked to many gastrointestinal manifestations [7]. *H. pylori* infects approximately fifty percent of people worldwide and is considered the most prevalent human pathogen. Its association with peptic ulcer and gastric malignancy has been clearly demonstrated, largely attributed to different factors like VacA, CagA, and various outer membrane proteins that trigger signaling pathways promoting aberrant cell growth and malignant transformation of the gastric mucosa [8]. A significant rate of co-infection has been reported between *H. pylori* and *Entamoeba histolytica*/*dispar*, with 55.5% of *H. pylori*-positive patients exhibiting concurrent intestinal amoebic infection [9]. Furthermore, a notable association was observed with the individuals positive for *H.pylori* being 1.19 times more likely to be positive for *E. histolytica* [10].

TNF-α, a pleiotropic cytokine, contributes to a wide range of biological processes, particularly inflammation, immune regulation, and cytotoxic activity. It plays a critical role in initiating host defense mechanisms against infectious agents. The TNF-α gene promoter is highly polymorphic, with at least 43 single-nucleotide polymorphisms (SNPs) identified, many of which have known minor allele frequencies. TNF-α expression and circulating levels have been linked to certain SNPs, including G-308A, G-238A, T-857C, and T-1031C, but published studies have yielded variable outcomes [11]. Abdel-Hafeez et al. [12] showed that increased TNF-α levels were significantly related to the severity of illness and diarrhea in patients infected with *Entamoeba histolytica*. TNF-α contributes to host defense by stimulating macrophages and neutrophils to release reactive oxygen species (ROS) and nitric oxide to eliminate *E*. *histolytica*, although these reactive molecules may also contribute to tissue injury [13]. Furthermore, findings from Davhana et al. [14] revealed that TNF-α promoter variants are associated with greater susceptibility to parasitic diseases, particularly those caused by *Entamoeba* species. During *H. pylori* infection, TNF-α release occurs locally and systemically, promoting inflammatory responses and predisposing to gastric disease [15].

While the clinical burden of *Helicobacter pylori* and the *Entamoeba* complex coinfection is increasingly acknowledged, particularly in endemic regions such as Egypt, there is a considerable debate concerning the genetic factors of the host that influence susceptibility and immune response. In particular, TNF-α promoter polymorphisms are understudied despite their established role in inflammatory response modulation. This study is designed to assess how TNF-α promoter polymorphisms may affect individual susceptibility to the *Entamoeba* complex infection considering both single infections and coinfection with *H. pylori.* Additionally, the prevalence of *H. pylori, E. dispar, E. histolytica, and E. moshkovskii* was determined and reported descriptively to provide a comprehensive epidemiological overview.

## Materials and methods

### Study design and setting

This cross-sectional molecular epidemiological study was conducted at the Diagnostic and Research Unit of Parasitic Diseases, Faculty of Medicine, Cairo University, Egypt (DRUPD). A total of 372 adult participants (≥ 18 years) were presented with gastrointestinal symptoms and were enrolled.

Participants were categorized into four equal groups (*n* = 93 each) based on PCR results: Group I, Helicobacter pylori–positive only; Group II, *Entamoeba* complex–positive only; Group III, co-infected with *H. pylori* and the *E.* complex; and Group IV, negative for both infections (healthy controls). Infection status was determined using standardized PCR-based assays. Participants with autoimmune diseases, immunodeficiency, inflammatory bowel disease, known genetic disorders, or those receiving immunosuppressive therapy were excluded. All eligible participants provided written informed consent before enrollment.

### Sample size calculation

The sample size calculation was calculated to detect differences in the distribution of TNF-α promoter polymorphisms between infected, co-infected, and uninfected. Based on the findings of Ahmed et al. [9], who reported a 55.5% co-infection rate among *H. pylori*-positive patients, we assumed a 50% prevalence of the TNF-α genetic polymorphism in the co-infected group and 30% prevalence in the control group. Using confidence level 95% (Z₁₋⍺/₂ = 1.96) and 80% power (Z₁₋ᵦ = 0.84), Participants per group were computed based on the following formula:

$$n={\left[\left({Z}_{1}-\alpha {/}_{2}\times \surd 2\overline{P }\left(1-\overline{P }\right)\right)+({Z}_{1-\beta }\times \surd ({P}_{1}\left(1-{P}_{1}\right)+{P}_{2}\left(1-{P}_{2}\right)\right]}^{2}\div {\left({P}_{1}-{P}_{2}\right)}^{2}$$

Where P₁ = 0.50, P₂ = 0.30, and P̄ = (P₁ + P₂)/2. The resulting total sample size required is 372 participants, with 93 participants in each of the four study groups.

### Ethical consideration

This study was conducted according to the principles of the Declaration of Helsinki (2024). The (IRB) of the National Hepatology and Tropical Medicine Research Institute, Cairo, Egypt, approved it (Serial No. 5–2025). All participants gave written informed consent following getting clear information about the aim of the study and procedures. Participation was completely voluntary, and confidentiality of all personal data was strictly maintained. The study posed minimal risk, limited to mild discomfort during stool sample collection.

### Sample collection and processing

Both macroscopic and microscopic examinations were carried out on stool samples. For microscopic evaluation, wet mount, staining with iodine, modified acid-fast staining, and trichrome staining were performed to diagnose *Entamoeba* species that may not be easily visualized by routine methods [16], and those positive for other intestinal parasites were excluded from analysis.

### DNA extraction

Stool specimens (200 mg) were used for DNA extraction after ten freeze–thaw cycles (liquid nitrogen and 95 °C) [17]. DNA was extracted using the QIAamp DNA Stool Mini Kit (Qiagen, Hilden, Germany; Cat. No. 51604) according to the manufacturer’s instructions. An extraction negative control (nuclease-free water, Thermo Fisher Scientific, USA; Cat. No. R0581) was processed in parallel with each batch to monitor for contamination. DNA integrity was confirmed using 1% agarose gel electrophoresis (UltraPure™ Agarose, Invitrogen™, Thermo Fisher Scientific, USA; Cat. No. 16500–500), and concentration and purity were measured using a NanoDrop™ 2000/2000c spectrophotometer (Thermo Fisher Scientific). Extracted DNA was stored at − 20 °C until further analysis.

### *Entamoeba complex* identification by Multiplex PCR

Multiplex PCR (m-PCR) focusing on the small subunit rRNA gene was applied to DNA extracts to identify *Entamoeba histolytica*, *E. moshkovskii*, and *E. dispar*, as documented by El-Badry et al. [17] (Table 1). PCR reactions were performed in a total volume of 25 µL containing 12.5 µL DreamTaq™ PCR Master Mix (Thermo Fisher Scientific, Waltham, MA, USA, Cat. No. K1071), 1 µL of each primer (10 µM; synthesized and HPLC-purified by Invitrogen™, Thermo Fisher Scientific, USA), 2 µL of DNA template, and nuclease-free water (Thermo Fisher Scientific; Cat. No. R0581) to complete the final volume. Electrophoresis on a 1.5% UltraPure™ agarose gel (Invitrogen™, Thermo Fisher Scientific; Cat. No. 16500–500), stained with ethidium bromide (Sigma-Aldrich, St. Louis, MO, USA; Cat. No. E1510), separated the PCR products, which were visualized under ultraviolet (UV) illumination. Expected band sizes were 167 bp (*E. histolytica*), 579 bp (*E. moshkovskii*), and 753 bp (*E. dispar*) [18]. Positive controls from previously confirmed species DNA archived in our laboratory (DRUPD), Faculty of Medicine, Cairo University, Egypt, were used to confirm assay specificity and amplification accuracy.

**Table 1:** Primers, PCR cycling, and expected fragment size

**Primers name**	**Primers**	**PCR** cycling	**Expected fragment size (bp)**
**EntaF**	**F:** 5'-ATGCACGAGAGCGAAAGCAT-3`	Cycles: 35, Denaturation: 94 °C, 1 min, Annealing: 58 °C, 1 min, Extension: 72 °C, 80 s	*E. histolytica*: 167 bp, *E. dispar*:753 bp,*E. moshkovskii* – 579 bp
**EhR**	**R:** 5'-GATCTAGAAACAATGCTTCTCT-3`		
**EdR**	**R:** 5'-CACCACTTACTATCCCTACC-3`		
**EmR**	**R:** 5'-TGACCGGAGCCAGAGACAT-3`		
**UreA**	**F1:** 5'-ATATTATGGAAGAAGCGAGAGC-3'	Cycles: 35 Denaturation: 94 °C, 1 min, Annealing: 57 °C, 1 min Extension: 72 °C, 1.5 min	293 bp
	**R1:** 5'- ATGGAAGTGTGAGCCGATTTG-3'		
	**F2:** 5'- CATGAAGTGGGTATTGAAGC-3'		200 bp
	**R2:** 5'- AAGTGTTGAGCCGATTTGAACCG-3'		
**CagA**	**F1:** 5'- GGAACCCTAGTC AGTAATGGGTT-3'	Cycles: 35, Denaturation: 94 °C, 15 s Annealing: 55 °C, 30 s Extension: 72 °C, 30 s	550 bp
	**R1:** 5'- GCTTTAGCTTCTGATACCGCTTGA-3'		
	**F2:** 5'- CCAATAACAATAATAATGGACTCAA-3'	Cycles: 35, Denaturation: 98 °C, 10 s, Annealing: 63 °C, 30 s, Extension: 72 °C, 30 s	
	**R2:** 5'- AATTCTTGTTCCCTTGAAAGCCC-3'		
**TNF-α 1031 T/C**	**F:** 5'-GGGGAGAACAAAAGGATAAG-3` **R:** 5'-CCCCATACTCGACTTTCATA-3**`**	Cycles: 30, Denaturation: 95 °C, 30 s, Annealing: 55 °C, 30 s, Extension: 72 °C, 30 s	264 bp
**TNF-α 308 G/A**	**F:** 5'- AGGCAATAGGTTTTGAGGGCCAT-3` **R:** 5'-ACACTCCCCATCCTCCCTGCT-3**`**	Cycles: 35, Denaturation: 94 °C, 30 s, Annealing: 60 °C, 30 s, Extension: 72 °C, 30 s	117 bp

### *H. pylori* n-PCR

Molecular screening of *Helicobacter pylori* infection by Nested PCR (n-PCR) amplification of the ureA gene was initially performed. To confirm *H. pylori* species identity and assess virulence, a second PCR was conducted to detect the cytotoxin-associated gene A (cagA), using specific primers (Table 1) [19, 20]. PCR reactions were performed using DreamTaq™ DNA Polymerase (Thermo Fisher Scientific, Cat. No. EP0702) with primers synthesized by Invitrogen™ (Thermo Fisher Scientific). PCR products were electrophoresed on 1.5% agarose gels stained with ethidium bromide and visualized under UV illumination. Expected product sizes were 200 bp for the ureA gene and 550 bp for the cagA gene. Each nested PCR run included positive controls comprising genomic DNA from reference *H. pylori* strain NCTC 11637 (ureA-positive) and a cagA-positive clinical isolate (previously confirmed by sequencing at our laboratory) alongside the negative controls (nuclease-free water, Cat. No. R0581) to ensure amplification accuracy, species identity confirmation, and exclusion of contamination.

### TNF-α Genotyping

DNA extraction for SNP genotyping was performed on 200 mg of fecal material according to standard protocols. PCR–RFLP was employed on the extracted DNA to detect TNF-α promoter polymorphisms at − 1031 T/C and − 308 G/A. Amplification of the target loci was conducted using the specific primers listed in Table 1, with DreamTaq™ DNA Polymerase (Thermo Fisher Scientific, Waltham, MA, USA, Cat. No. EP0702) and primers synthesized by Invitrogen™ (Thermo Fisher Scientific, USA). At 37 °C, the PCR products were subjected to restriction digestion using BbsI (Cat. No. ER1011) and NcoI (Cat. No. ER0571) restriction enzymes (Thermo Fisher Scientific, Waltham, MA, USA) according to the manufacturer’s instructions, as described previously [21, 22]. The digested fragments were separated on 3% UltraPure™ agarose gels (Invitrogen™, Thermo Fisher Scientific, Cat. No. 16500–500), stained with ethidium bromide (Sigma-Aldrich, St. Louis, MO, USA, Cat. No. E1510) and visualized under ultraviolet light. Each PCR–RFLP assay included a known TNF-α genotype DNA as a positive control and nuclease-free water (Cat. No. R0581) as a negative control to verify accuracy and exclude contamination.

### Statistical analysis

It was carried out by using IBM SPSS Statistics, version 28.0 (IBM Corp., Armonk, NY, USA). Mean ± SD were used to summarize continuous variables, and frequencies and percentages were used to present categorical variables. Chi-square analysis examined the relationship of TNF-α promoter polymorphic profiles to concurrent infection with *H. pylori* and *E. histolytica*. We calculated odds ratios with 95% confidence intervals to summarize the associations. Differences in allele and genotype frequencies across study groups (co-infected, single-infected, and control) were evaluated using Fisher’s exact test. The relationship between parametric and non-parametric variables was investigated by Spearman’s correlation coefficient. Findings were deemed significant when the *P* value was < 0.05.

## Results

### Baseline characteristics of the participants

This research enrolled 372 individuals. Their mean age was 38.3 ± 13.4 years, with ages spanning 18 to 75 years. Among them, 61.0% were male and 39.0% were female. The majority resided in urban areas (54.0%), and 60.2% reported consuming tap water as their primary water source.

### Prevalence, genotypes, and risk factors of the Entamoeba complex

Out of the ninety-three stool samples diagnosed as the *Entamoeba* complex by microscopic examination, Figure S1. Multiplex PCR revealed that *E. moshkovskii* was the prevalent species, detected in fifty cases (53.8%), *E. dispar* in 27 cases (29.0%), and *E. histolytica* in 16 cases (17.2%), Figure S2.

Mixed infections were also observed in four cases (*E. histolytica* & *E. dispar*), additionally in 15 cases (*E. dispar* & *E. moshkovskii*). No mixed infection of *E. histolytica* and *E. moshkovskii* was detected. As shown in Table 2, a significant association was found between socio-economic status and infection with the *E. histolytica* complex. The likelihood of infection was twice as high among males (OR = 2.0; 95% CI: 1.12–3.59; *P* = 0.019), and younger participants demonstrated higher susceptibility (OR = 4.5; 95% CI: 0.70–8.29; *P* = 0.020). Furthermore, consumption of tap water showed a strong association with the infection (OR = 4.1; 95% CI: 2.19–7.48; *P* < 0.0001). Infection showed significant associations with symptoms including diarrhea, colic, vomiting, loss of appetite, and fever. The prevalences of *E. histolytica*, *E. moshkovskii*, and *E. dispar* differed across multiple risk factors, including age, sex, residence, water source, and animal contact (Table 3). *E. histolytica* and *E. dispar* infections were observed more frequently among older individuals compared with *E. moshkovski* (*P* = 0.045). *E. histolytica* and *E. moshkovskii* showed higher prevalence in males (81.3% and 62.0%, respectively), whereas *E. dispar* was more frequently detected in females (40.7%), a statistically significant difference (*P* = 0.027). Regarding residence, *E. moshkovskii* was predominant in urban areas (70.0%), whereas in rural areas both *E*. *dispar* and *E. histolytica* were more common (25.0% and 25.9%, respectively; *P* = 0.0001). Water sources had a significant impact, as *E. histolytica* and *E. dispar* were more common among tap water consumers (93.8% and 92.6%, respectively; *P* = 0.0001). Participants with a history of animal contact showed a markedly higher percentage of infection with *E. histolytica* (62.5%) and *E. dispar* (70.4%), a statistically significant difference (*P* < 0.0001). Regarding gastrointestinal manifestations, diarrhea, abdominal pain, and loss of appetite were significantly associated *E. histolytica*, *E. moshkovskii,* and *E. dispar.* To clarify the role of co-infections, all *Entamoeba*-positive cases were tested for *H. pylori*, revealing both mono- and co-infections, with a subset of *E. dispar*– and *E. moshkovskii*–infected individuals also positive for *H. pylori*.

**Table 2:** Risk factors for *Entamoeba complex*-infected compared with controls

Variables	Infected with *E. complex* (*n* = 93)	Controls (*n* = 93)	OR &(95% CI)	*P*-value
Age (Mean ± SD)	34.4 ± 11.7	38.9 ± 14.4	4.5 (0.70- 8.29)	0.020^*^
Sex (M/F)	55/38	39/54	2.0 (1.12- 3.59)	0.019^*^
Residence (Urban/Rural)	46/47	42/51	1.2(0.67–2.11)	0.557
Water source (Tap/Filter)	66/27	35/58	4.1 (2.19- 7.48)	*P* < 0.0001^*^
Animal contact (Yes/No)	36/57	54/39	0.46 (0.25- 0.82)	0.008^*^
Diarrhoea (Yes/No)	40/53	7/86	9.3 (3.87- 22.19)	*P* < 0.0001^*^
Colic (Yes/No)	54/39	21/72	4.7 (2.51- 8.98)	*P* < 0.0001^*^
Vomiting (Yes/No)	0/93	1/92	0.33 (0.01- 8.20)	0.498
Loss of appetite (Yes/No)	3/90	49/44	0.03 (0.009–0.101)	*P* < 0.0001^*^
Fever (Yes/No)	0/93	24/69	0.02 (0.001- 0.254)	0.003^*^

* significant difference at *P*<0.05

**Table 3:** *Entamoeba* complex with studied variables

Variables	*E. histolytica* (*n* = 16)	*E. dispar* (*n* = 27)	*E. moshkovskii* (*n* = 50)	*P*-value
Age (Mean ± SD)	40.8 ± 10.9	34.5 ± 12.6	32.8 ± 10.1	0.045^*^
Sex (M/F)	13/3	11/16	31/19	0.027^*^
Residence (Urban/Rural)	4/12	7/20	35/15	0.0001^*^
Water source (Tap/Filter)	15/1	25/2	26/24	0.0001^*^
Animal contact (Yes/No)	10/6	19/8	7/43	*P* < 0.0001^*^
Diarrhoea (Yes/No)	16/0	11/16	13/37	*P* < 0.0001^*^
Abdominal pain (Yes/No)	16/0	17/10	21/29	0.0002^*^
Vomiting (Yes/No)	0/16	0/27	0/50	–
Loss of appetite (Yes/No)	0/16	3/24	0/50	0.023^*^
Fever (Yes/No)	0/16	0/27	0/50	^−−^

* significant difference at *P*<0.05

### *H. pylori* Infection: Prevalence and associated factors

By using PCR*, H. pylori* infection was diagnosed in 93 stool samples, of which 41 cases (44.1%) were positive for the CagA virulence gene (Figure S3). A strong association was observed between *H. pylori* infection and male gender consuming tap water, while no statistically significant associations were observed with age or residence. Regarding clinical manifestations, infected individuals were more likely to report abdominal pain (*P* = 0.006) (Table 4).

**Table 4:** Risk factors for *H. pylori-*infected and healthy controls

Variables	Infected with *H.pylori* (*n* = 93)	Healthy control (*n* = 93)	OR& (95% CI)	*P*-value
Age (Mean ± SD)	40.7 ± 14.0	38.9 ± 14.4	−1.8 (−5.91–2.39)	0.389
Sex (M/F)	63/30	39/54	2.9 (1.59–5.29)	0.0005^*^
Residence (Urban/Rural)	53/40	42/51	1.6 (0.90–2.87)	0.108
Water source (Tap/Filter)	69/24	35/58	4.8 (2.55–8.91)	*P* < 0.0001^*^
Animal contact (Yes/No)	32/61	54/39	0.38 (0.21- 0.69)	0.001^*^
Diarrhoea (Yes/No)	1/92	7/86	0.13 (0.02–1.11)	0.062
Abdominal pain (Yes/No)	7/86	21/72	0.28 (0.11–0.69)	0.006^*^
Vomiting (Yes/No)	1/92	1/92	1.00 (0.06–16.23)	1.00
Loss of appetite (Yes/No)	37/56	49/44	0.59 (0.33–1.06)	0.078
Fever (Yes/No)	19/74	24/69	0.74 (0.37–1.47)	0.385

* significant difference at *P*<0.05

### Co-Infection by the *Entamoeba complex *and *H. pylori*

Univariate analysis identified factors linked to co-infection with the *Entamoeba* complex and *H. pylori* (Table 5). A significant association was found between co-infection status and male sex (OR = 4.2; 95% CI: 2.25–7.88; *P* < 0.0001). Participants who consumed tap water were also at higher risk of co-infection (OR = 2.3; 95% CI: 1.27–4.13; *P* = 0.005). Clinically, diarrhea (*P* < 0.0001) and abdominal pain (*P* = 0.0003) were observed significantly more often among co-infected participants compared with the control group.

**Table 5:** Risk factors for patients with Co-infection of the *Entamoeba* complex and *H.pylori* compared to controls

Variables	Co-infection of *Entamoeba complex* & *H.pylori* (*n* = 93)	Healthy control (*n* = 93)	OR (95% CI)	*P*-value
Age (Mean ± SD)	36.0 ± 11.7	38.9 ± 14.4	2.9 (−0.86–6.66)	0.129
Sex (M/F)	70/23	39/54	4.2 (2.25–7.88)	P < 0.0001^*^
Residence (Urban/Rural)	50/43	42/51	1.4 (0.79–2.51)	0.241
Water source (Tap/Filter)	54/39	35/58	2.3 (1.27–4.13)	0.005^*^
Animal contact (Yes/No)	42/51	54/39	0.59 (0.33–1.06)	0.075
Diarrhoea (Yes/No)	46/47	7/86	12.0 (5.03–28.73)	P < 0.0001^*^
Abdominal pain (Yes/No)	45/48	21/72	3.2 (1.71–6.06)	0.0003^*^
Vomiting (Yes/No)	0/93	1/92	0.33 (0.01–8.20)	0.499
Loss of appetite (Yes/No)	0/93	49/44	0.005 (0.0003–0.0797)	0.0002^*^
Fever (Yes/No)	0/93	24/69	0.02 (0.0009–0.254)	0.004^*^

* significant difference at *P*<0.05

### TNF-α polymorphisms and infection with the *Entamoeba complex* and/or *H. pylori*

Analysis of TNF-α − 1031 T/C and TNF-α − 308G/A polymorphisms showed no significance between participants infected with the *Entamoeba* complex and controls. In contrast, *H. pylori* infection was significantly associated with both polymorphisms (Table 6, Figure S4). The TT genotype and T allele of TNF-α − 1031 T/C were most frequent among controls and were associated with a protective effect against *H. pylori* infection (CC genotype: OR = 2.0, 95% CI: 1.12–3.64, *P* = 0.019; C allele: OR = 2.5, 95% CI: 1.34–4.69, *P* = 0.004). Conversely, the TT genotype and T allele occurred more frequently among participants with *H. pylori* infection, and both demonstrated significant associations with infection (*P* = 0.0006 and *P* = 0.006). Assessing TNF-α − 308G/A revealed that the GA genotype occurred more frequently in controls, suggesting a protective role against *H. pylori* (OR = 1.89, 95% CI: 1.06–3.38; *P* = 0.030). In contrast, the AA genotype (homozygous mutant) was significantly more common among infected participants (*P* = 0.036). The frequencies of TNF-α genotypes and alleles across single and co-infection groups are presented in Table 7.

**Table 6:** TNF*-α* Polymorphisms in the *Entamoeba* Complex and *H. pylori* Infections

SNP	Controls (*n* = 93)	*Entamoeba complex* (*n* = 93)	*H. pylori* (*n* = 93)	*Entamoeba* complex & controls		*H. pylori* & controls	
				**OR& (95% CI)**	*P*** value**	**OR & (95% CI)**	*P*** value**
**TNF-α -1031 T/C (Allele and genotypes)**							
TT	67 (72.0%)	71 (76.3%)	52 (55.9%)	0.81 (0.43–1.53)	0.519	2.0 (1.12- 3.64)	0.019^*^
TC	12 (13.0%)	10 (10.8%)	7 (7.5%)	1.2 (0.51–2.84)	0.664	1.7 (0.68- 4.35)	0.253
CC	14 (15.0%)	12 (12.9%)	34 (36.6%)	1.2 (0.53–2.63)	0.684	0.30 (0.15–0.59)	0.0006^*^
T	146 (78.5%)	152 (81.7%)	111 (59.7%)	0.83 (0.41–1.67)	0.593	2.5 (1.34–4.69)	0.004^*^
C	40 (21.5%)	34 (18.3%)	75 (40.3%)	1.3 (0.64–2.58)	0.480	0.42 (0.23–0.79)	0.006^*^
**TNF-α-308 G/A (Allele and genotypes)**							
GG	37 (39.8%)	30 (32.3%)	40 (43.0%)	1.4 (0.79–2.53)	0.239	0.88 (0.50–1.55)	0.667
GA	43 (46.2%)	52 (55.9%)	29 (31.2%)	0.67 (0.38–1.17)	0.158	1.89 (1.06–3.38)	0.030^*^
AA	13 (14.0%)	11 (11.8%)	24 (25.8%)	1.2 (0.52–2.73)	0.674	0.46 (0.23–0.95)	0.036^*^
G	117 (62.9%)	112 (60.2%)	109 (58.6%)	1.1 (0.64–2.01)	0.663	1.2 (0.67–2.09)	0.562
A	69 (37.1%)	74 (39.8%)	77 (41.4%)	0.88 (0.49–1.56)	0.663	0.85 (0.48–1.50)	0.562

* significant difference at *P*<0.05

**Table 7:** TNF*-α* in *E. histolytica*–*H. pylori* Co-Infected Patients

SNP	Co-infection with *Entamoeba.* complex and *H. pylori* (*n* = 93)	*Entamoeba* complex* (n* = *93)*	*H. pylori* (*n* = 93)	*Entamoeba* complex and *H. pylori* vs *Entamoeba complex (n* = 93)		*Entamoeba* complex and *H. pylori* vs *H.pylori* (*n* = 93)	
				**OR& (95% CI)**	***P* value**	**OR & (95% CI)**	***P* value**
TT	65 (69.9%)	71 (76.3%)	52 (55.9%)	0.74 (0.39–1.38)	0.340	1.8 (1.02–3.28)	0.041^*^
**TNF-α −1031 T/C (Allele and genotypes)**							
TC	12 (12.9%)	10 (10.8%)	7 (7.5%)	1.2 (0.51–2.84)	0.662	1.7 (0.68–4.35)	0.253
CC	16 (17.2%)	12 (12.9%)	34 (36.6%)	1.4 (0.63–2.99)	0.429	0.35 (0.18- 0.68)	0.001^*^
T	142 (76.3%)	152 (81.7%)	111 (59.7%)	0.69 (0.35- 1.38)	0.699	2.1 (1.15–3.88)	0.016^*^
C	44 (23.7%)	34 (18.3%)	75 (40.3%)	2.1 (1.01- 4.44)	0.048^*^	0.47 (0.26- 0.87)	0.016^*^
**TNF-α−308 G/A (Allele and genotypes)**							
GG	29 (31.2%)	30 (32.3%)	40 (43.0%)	0.95 (0.53–1.73)	0.879^*^	0.59 (0.33- 1.06)	0.079^*^
GA	48 (51.6%)	52 (55.9%)	29 (31.2%)	0.72 (0.41- 1.26)	0.255	2.4 (1.35- 4.29)	0.002^*^
AA	16 (17.2%)	11 (11.8%)	24 (25.8%)	1.5 (0.68- 3.33)	0.317	0.58 (0.29- 1.16)	0.124
G	106 (57.0%)	112 (60.2%)	109 (58.6%)	0.88 (0.50- 1.55)	0.667	0.92 (0.53- 1.62)	0.775
A	80 (43.0%)	74 (39.8%)	77 (41.4%)	1.1 (0.64–1.99)	0.667	1.1 (0.62- 1.90)	0.775

* significant difference at *P*<0.05

## Discussion

Mixed infections with gastrointestinal pathogens are a significant public health concern, particularly in developing countries [23]. Recent studies have examined the prevalence of intestinal protozoa and *Helicobacter pylori* across different populations [24, 25], highlighting that co-infections can modulate the intestinal microbiome and host immune-inflammatory pathways, sometimes exerting anti-inflammatory effects [26, 27]. Accordingly, this study investigated the role of the pro-inflammatory cytokine TNF-α in *Entamoeba* complex infection and its potential association with susceptibility to co-infection with *H. pylori.*

To minimize misattribution of gastrointestinal symptoms, other intestinal parasitic infections were excluded during initial screening. Moreover, the *H. pylori* infection status of *Entamoeba*-positive participants was assessed, confirming that a subset of *E. dispar*– and *E. moshkovskii–*infected individuals were co-infected with *H. pylori*. This co-infection may partly explain the observed gastrointestinal manifestations, given that *E. dispar* is generally considered nonpathogenic.

In this study, among 93 stool samples initially diagnosed with *Entamoeba* complex by microscopic examination, molecular characterization showed that *E. moshkovskii* was the most common species (53.8%), followed by *E*. *dispar* (29.0%) and *E. histolytica* (17.2%). Mixed infections were also detected*.* These findings align with those of Samie et al. [28], who reported a higher prevalence of *E. moshkovskii* compared with other Entamoeba species.

In the current study, *Entamoeba* complex infection was significantly associated with younger age and male sex. These are consistent with other previous studies showing higher susceptibility among young adults and males [29–31]. However, Gebretsadik (2016) reported a higher prevalence among females [32], highlighting geographic and population-related differences. Tap water consumption was strongly associated with increased infection prevalence, in agreement with Atabati et al. [33], particularly in settings with inadequate sanitation. Infection was also more frequent among individuals with animal contact, supporting the role of animals as potential reservoirs and sources of zoonotic transmission. Additionally, *Entamoeba* complex infection was significantly associated with gastrointestinal symptoms, including diarrhea, abdominal pain, vomiting, loss of appetite, and fever, emphasizing its clinical relevance.

In this study, species-specific differences within the *Entamoeba* complex were observed across demographic and environmental factors. *E. histolytica* and *E. dispar* were more frequent in older individuals, while sex-related differences were evident, with *E. histolytica* and *E. moshkovskii* predominating in males and *E. dispar* being more common in females. Residence also influenced species distribution, as *E. moshkovskii* was mainly detected in urban populations, whereas *E. histolytica* and *E. dispar* were more prevalent in rural settings. Tap water consumption and animal contact were strongly associated with *E. histolytica* and *E. dispar* infections, supporting the role of environmental exposure and potential zoonotic transmission. Clinically, all three *Entamoeba* species were significantly associated with gastrointestinal symptoms, particularly diarrhea, abdominal pain, and loss of appetite. The association between tap water consumption and infection with *E. histolytica* and *E. dispar*, but not *E. moshkovskii*, may reflect differences in transmission ecology, as *E. moshkovskii* can persist in diverse environmental reservoirs such as soil and sewage, potentially explaining its comparable detection among individuals consuming tap or filtered water.

In this study, *H. pylori* infection was detected by PCR, with a substantial proportion of strains carrying the CagA virulence gene, which is associated with increased pathogenicity. This finding is consistent with previous reports from Egypt [34], although higher CagA prevalence has been described in patients with gastric adenocarcinoma in other populations [35]. *H. pylori* infection was more common among males, in agreement with earlier studies [36], a difference that may reflect variations in lifestyle and environmental exposure. Tap water consumption was also strongly associated with infection, supporting evidence that contaminated water represents an important transmission route [37]. In contrast, age and residence were not significantly associated with infection. Clinically, infected participants more frequently reported abdominal pain, consistent with previous observations [38].

Previous studies have shown that *H. pylori* is frequently associated with intestinal parasitic infections, and untreated co-infections may result in substantial health risks [39–42]. Limited research has examined the specific relationship between *H. pylori* and the *Entamoeba* complex. Co-infection appears to be strongly influenced by socio-environmental factors, including poor hygiene and unsafe water sources [39]. In the present study, co-infection was more common among males, consistent with earlier findings [43], and was significantly associated with tap water consumption, supporting the role of water quality in transmission. Other factors, such as age, residence, and animal contact, showed no significant associations. Clinically, co-infected patients more frequently report diarrhea and abdominal pain, in agreement with previous reports indicating more pronounced gastrointestinal symptoms in co-infected individuals [43, 44].

Variants involving transitions have been reported in the TNF-α promoter at positions − 237, − 307, − 375, − 856, − 862, and − 1031. Of these, the − 307 and − 237 loci are the most frequently studied in relation to *H. pylori* infection, particularly with respect to susceptibility and disease progression [45]. Elevated TNF-α production has also been linked to the − 1031 SNP, which is reported at higher frequencies in African populations [46].

The distribution of TNF-α − 1031 T/C and − 308G/A polymorphisms did not differ significantly between *Entamoeba complex*–infected participants and controls; however, *H. pylori* infection showed clear associations with both loci. For the − 1031 T/C variant, the TT genotype and T allele were more frequent among healthy controls, suggesting a protective effect, whereas the CC genotype and C allele were associated with increased susceptibility to *H. pylori*. Similarly, for the − 308G/A polymorphism, the GA genotype was more prevalent in controls, indicating a potential protective role, while the AA genotype was more common among infected individuals. These findings suggest that TNF-α promoter polymorphisms may modulate host susceptibility to *H. pylori* infection, possibly through effects on inflammatory response intensity. Functionally, TNF-α promoter polymorphisms can influence cytokine expression and host inflammatory responses. The − 308 A allele is associated with enhanced TNF-α transcription and stronger gastric inflammation [21, 22], whereas the − 1031 C allele may reduce promoter activity and cytokine secretion [14]. These variants may therefore modulate the intensity of the immune response to *H. pylori*, contributing to differences in infection susceptibility and disease severity observed in this study [11, 46].

## Limitations

A key limitation of this study is the use of fecal DNA for host SNP analysis, which generally yields lower-quality and more fragmented DNA compared to blood-derived samples. However, fecal DNA was selected to ensure direct infection–genotype correlation from the same specimens and to minimize participant burden, as only stool collection was IRB-approved. To mitigate potential limitations, a high-yield QIAamp extraction method was applied, DNA quality was verified, short PCR–RFLP amplicons (< 300 bp) were used, and complete concordance was achieved in replicate genotyping. Future validation using paired blood samples is recommended.

## Conclusion

In this Egyptian study (*n* = 372), co-infection with *Helicobacter pylori* and the *Entamoeba* complex was common and associated with Gastrointestinal symptoms; male sex and tap-water use were key risk factors. *E. moshkovskii* predominated (53.8%), and 44.1% of *H. pylori* were *cagA*-positive. TNF-α promoter variants were associated with *H. pylori*: − 1031 T/TT and − 308 GA were overrepresented in controls, whereas − 308 AA was more frequent in cases; no association was seen with *Entamoeba* alone. These loci warrant further investigation as biomarkers, and improvements in water quality/sanitation, plus targeted molecular screening, may reduce the burden.

## Supplementary Information

Supplementary Material 1

## Data Availability

The datasets generated and analyzed during this study are available from the corresponding author upon reasonable request.

## Abbreviations

bp: Base pair
CagA: Cytotoxin-associated gene A
DNA: Deoxyribonucleic acid
DRUPD: Diagnostic and Research Unit of Parasitic Diseases
*E. dispar*: *Entamoeba dispar*
*E. histolytica*: *Entamoeba histolytica*
*E. moshkovskii*: *Entamoeba moshkovskii*
*H. pylori*: *Helicobacter pylori*
n-PCR: Nested polymerase chain reaction
PCR: Polymerase chain reaction
PCR-RFLP: Polymerase chain reaction–restriction fragment length polymorphism
SPSS: Statistical Package for the Social Sciences
SD: Standard deviation
SNPs: Single-nucleotide polymorphisms
TNF-α: Tumor necrosis factor-alpha
