# Supplementary material for: Role of TNF-α genetic variants in coinfection with Helicobacter pylori and the Entamoeba complex: a cross-sectional study
Source: BMC Microbiol. 2026 Apr 16;26:378. doi: 10.1186/s12866-026-04964-2 (PMC13088695; doi:10.1186/s12866-026-04964-2)
Supplement: Supplementary file 1 — Supplementary Material 1 [file 12866_2026_4964_MOESM1_ESM.docx]

**Supplementary file**

**
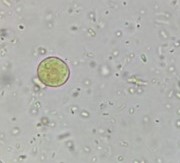
**

**Figure 1S:** *Entamoeba complex* cyst stained with iodine


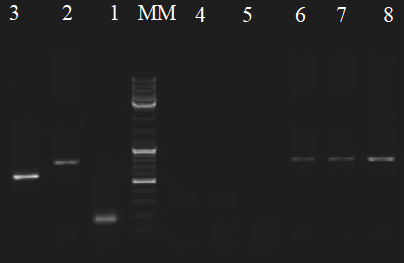


**Figure 2S**: PCR amplification of *Entamoeba*-specific. Lane MM: DNA marker of 100 bp molecular weight. Lane 1: positive *E. histolytica* sample at 166 bp. Lanes 2, 6,7 and 8: positive *E.* *dispar* samples at 752 bp. Lane 3: positive *E. moshkovski* samples at 580 bp. Lanes 4 and 5: negative samples.


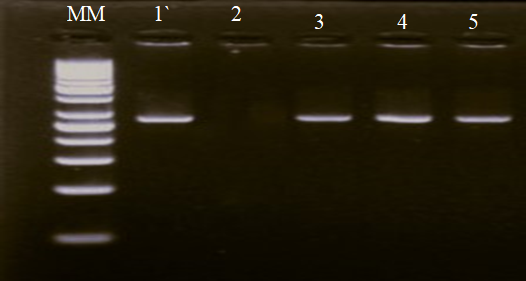


**Figure 3S**: PCR amplification of genus-specific CagA for *H. pylori*. Lane MM contains a 100 bp ladder, Lane 1: Positive control, Lane 2: Negative control, Lanes 3, 4, and 5 contain *H. pylori*-specific PCR products at 550 bp isolated from stool samples.


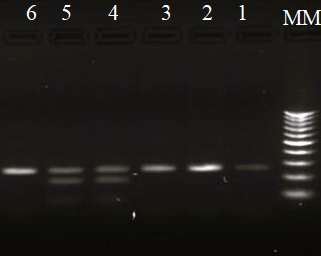


**Figure 4S**: Agarose gel electrophoresis showing the PCR-RFLP product of TNF- α -1031 after digestion with BbsI restriction enzyme. Lane MM contains a 100 bp ladder, Lane 1,2, 3, and 6: TT genotype was achieved at 251 bp, Lanes 4, 5: TC genotype was achieved with bands of 251,180, and 71 bp sizes
